# Implementation Strategies for Quality Improvement in Palliative Care: A Scoping Review

**DOI:** 10.1111/hex.14151

**Published:** 2024-07-26

**Authors:** Yunyun Dai, Barbara A. Daveson, Luyu Gan, Jinfeng Ding, Yongyi Chen, Claire E. Johnson

**Affiliations:** ^1^ Faculty of Science, Medicine and Health University of Wollongong Wollongong New South Wales Australia; ^2^ School of Nursing Guilin Medical University Guilin Guangxi China; ^3^ Palliative Care Outcomes Collaboration, Faculty of Science, Medicine and Health University of Wollongong Wollongong New South Wales Australia; ^4^ Xiangya School of Nursing Central South University Changsha Hunan China; ^5^ Hunan Cancer Hospital The Affiliated Cancer Hospital of Xiangya School of Medicine, Central South University Changsha Hunan China; ^6^ Palliative Aged Care Outcomes Program, Faculty of Science, Medicine and Health University of Wollongong Wollongong New South Wales Australia

**Keywords:** implementation strategies, palliative care, person‐centered outcomes, quality improvement program

## Abstract

**Background:**

Quality improvement (QI) programs based on person‐centred outcome measures (PCOMs) play an important role in promoting optimal palliative care. However, routine use of PCOMs has been slow and difficult to implement, including within QI programs.

**Objective:**

This study aimed to identify implementation strategies that support the implementation of PCOMs as routine practice in hospital‐based palliative care, as well as the implementation theories, models and frameworks (TMFs) guiding the design of these implementation strategies.

**Methods:**

A scoping review was conducted in accordance with the Joanna Briggs Institute (JBI) Scoping Review framework. Four databases (Medline, CINAHL, Scopus and PubMed) were systematically searched for literature published between 1 January 1990 and 8 March 2024.

**Results:**

One hundred and fifteen unique implementation strategies, identified from 11 included studies, were mapped onto the 73 Expert Recommendations for Implementing Change (ERIC) discrete implementation strategies, covering 52% of the ERIC strategies. The most commonly used categories were *train and educate stakeholders,* and *support clinicians*, followed by *develop stakeholder interrelationships* and *use evaluation and iterative strategies*. Three key themes emerged: w*hat to do*; *how to do it*; and *who to do it with*. Only four studies employed TMFs to guide the design of the implementation strategies in this review.

**Conclusions:**

To promote the implementation of PCOM‐based QI programs, strategies should be developed based on identified/potential barriers and facilitators by using rigorous TMFs. The components of the implementation strategies must be reported transparently and consistently to enable replication and measurement in future research and practice.

**Patient and Public Contribution:**

This scoping review does not directly involve patients or the general public in its design or execution. However, it is part of an implementation study aimed at integrating the Palliative Care Outcome Collaboration (PCOC) model into routine clinical practice at a cancer hospital in China. Before the formal implementation, palliative care professionals from this hospital highlighted the need for a comprehensive analysis of existing evidence to support the effective adoption of the PCOC model in their specific clinical setting.

## 1 Introduction

Palliative care aims to improve the quality of life for people with life‐limiting diseases and their families/carers by addressing their physical, emotional, social and spiritual needs [1]. High‐quality palliative care results in better symptom management, increased patient satisfaction, improved quality of life and reduced healthcare costs [2, 3].

Person‐centred care, which prioritizes the preferences, values and needs of patients and their families/carers, has been recognized as an essential component of high‐quality palliative care [4]. Person‐centred outcome measures (PCOMs) are standardized and validated questionnaires that include both patient‐reported and proxy‐reported outcome measures [5]. These tools provide healthcare providers with a way of identifying, measuring and comparing the patient's most important health‐related concerns [6]. It has been widely recognized that routine use of PCOMs in palliative care clinical practice may promote the quality of palliative care both at the individual and organizational level through better communication, identification and monitoring of needs and enhanced care planning, decision‐making and care delivery [7, 8]. Quality improvement (QI) programs involving the routine use of PCOMs were widely used to drive enhancements in healthcare quality [5, 7].

Despite the effectiveness and benefits, the routine use of PCOM‐based QI programs in clinical practice is lagging due to many challenges, such as time constraints of healthcare professionals, inadequate resources, lack of training/knowledge, negative attitudes, fear of added work and limited support from colleagues and managers [9, 10]. Implementation science, a scientific study of methods aimed at promoting the systematic uptake of research findings and other evidence‐based interventions into routine practice, emerged in the last decade [11]. Implementation strategies are regarded as key to closing the evidence–practice gap in the field of implementation science, as they constitute the ‘how to’ components and steps to improve the speed, quantity and quality of implementation [12, 13]. Currently, some encouraging implementation strategies, such as training, peer support, champions/facilitators, regular feedback and information technology infrastructure have been developed to support the implementation of QI programs for palliative care [9, 14]. However, these strategies have not yet been systematically summarized [15], and it was also been found difficult to replicate, generalize or compare the effectiveness of these strategies. This is due to the common lack of operational manuals, poor descriptions, different definitions or inconsistent terminology caused by variations in the underpinning implementation theories, models and frameworks (TMFs) [13, 16]. This ‘secondary’ research‐to‐practice gap makes it difficult to advance the implementation knowledge, and results in difficulty in the rollout of the PCOM‐based QI programs.

To enable replication, evidence synthesis and broader implementation of QI programs based on the routine use of PCOMs in palliative care, implementation strategies must be described fully and precisely using a standardized language. We selected the Expert Recommendations for Implementing Change (ERIC) taxonomy for this purpose due to its comprehensive and consensus‐based development by experts in implementation and clinical practice, as well as its widespread use in the field of implementation science [17]. The ERIC framework comprises nine categories covering 73 implementation strategies (the categories and the strategies of the ERIC framework are presented in Supporting Information S1: Table 3), compiled into taxonomies with detailed definitions that facilitate the translation of knowledge into routine care [17]. Additionally, the ERIC framework employs the Go‐zone quadrant analysis to rank the importance and feasibility of implementation strategies [18] (Supporting Information S1: Table 3). This analysis categorizes strategies into four quadrants based on expert ratings:

- Quadrant I: High importance and high feasibility.
- Quadrants II: High feasibility but lower importance.
- Quadrant III: Low importance and low feasibility.
- Quadrants IV: High importance but lower feasibility.

This categorization assists decision‐makers in effectively prioritizing strategies, ensuring that resources are allocated to the most impactful and achievable interventions.

Therefore, this review aims to explore the breadth of strategies used to support the implementation of PCOM‐based QI programs by using the ERIC language and to identify potential gaps in how palliative care researchers and practitioners articulate their design. Our scoping review addressed two questions:

1. What types of strategies have been used to support the implementation of QI programs based on PCOMs within palliative care?
2. What TMFs have been employed to guide the design of implementation strategies for PCOM‐based QI programs in hospital‐based palliative care?

By answering these questions, this review will provide a comprehensive overview of the existing implementation strategies, highlighting best practices and identifying areas where further research and development are needed. This will help bridge the evidence–practice gap, improve the quality of palliative care and guide future efforts in the systematic integration of PCOM‐based QI programs.

## 2 Methods

The scoping review was guided by the Joanna Briggs Institute (JBI) Scoping Review framework [19] and is reported in accordance with the Preferred Reporting Items for Systematic Reviews and Meta‐Analyses Scoping Review (PRISMA‐ScR) checklist [20]. The review protocol was registered on Open Science Framework (Registration DOI is https://doi.org/10.17605/OSF.IO/AQ9TR).

### 2.1 Eligibility Criteria

The ‘PCC’ mnemonic (Population, Concept, Context) was used as a guide to identify key concepts in the review and to construct clear eligibility criteria for inclusion [19]. We specified that the study should provide information relating to the implementation strategies of QI programs based on PCOMs (Concept) in hospital‐based palliative care practice (Context). We defined hospital‐based palliative care services as palliative care consult teams and inpatient palliative care units. We did not specify the population as multiple stakeholders could contribute to developing the implementation strategies, such as palliative care clinicians, psychologists, social workers, nutritionists, implementation science researchers and others involved in palliative care. All study designs were considered. The inclusion and exclusion criteria are presented in Table 1.

**Table 1:** The inclusion and exclusion criteria.

	Inclusion criteria	Exclusion criteria
Population	Not specified	Not specified
Concept	The implementation strategies of the quality improvement programs The quality improvement programs based on PCOMs	The development of a quality improvement program based on PCOMs The effectiveness of the quality improvement program based on PROMs, which did not include information on the implementation strategies or the effectiveness of the implementation strategies The barriers and enablers perceived by the stakeholders, which did not include information on the implementation strategies
Context	Hospital‐based palliative care practice settings (both adult and paediatric palliative care settings were included)	Other healthcare settings other than hospital‐based palliative care settings, such as community‐based palliative care settings and home‐based palliative care settings
Study	English language Primary study published in a peer‐reviewed journal All types of study designs	Published literature other than primary study, i.e., protocol, reviews, conference abstracts and books.

### 2.2 Data Sources and Search Strategy

Four databases (Medline, CINAHL, Scopus and PubMed) were systematically searched for literature published between 1 January 1990 and 12 October 2022, with an update extending to 8 March 2024. This timeline was chosen because PCOMs began to emerge in the literature in the late 1980s. Reference lists of the included literature and previous relevant reviews were manually checked. Grey literature was excluded.

We identified keywords for developing the search strategy based on previous reviews related to our topics [6, 10, 21, 22]. The Cochrane Library was used to find synonyms to ensure all the related terminology was included. We refined the search strategy with assistance from a university librarian to ensure it captured articles known to meet the search criteria and a reasonable number of relevant articles. The search strategy used in Medline is presented in Supporting Information S1: Table 1. Necessary modifications were made according to each database's specific requirements.

### 2.3 Study Selection

All studies identified from the databases were exported into the systematic review management software program Covidence [23], where duplicates were identified and removed. Title and abstract screening were performed independently by two reviewers (Y.D. and L.G.). Any discrepancies at this stage were resolved by discussion. Studies that potentially met the inclusion criteria were then advanced to full‐text review, also performed independently by Y.D. and L.G. Disagreements at this phase were resolved through a combination of discussion and consensus meetings involving Y.D., L.G., J.D. and C.E.J. We did not conduct a quality appraisal for the included studies in accordance with the guidance of the JBI scoping review framework.

### 2.4 Data Extraction

An Excel worksheet was developed before data extraction to document items of interest from included studies. Extracted data included authors/country/year, aim, study setting, study design, data collection method/participants, detailed information of the QI programs based on PCOMs (names, tools, raters and assessment frequency), the detailed information of the implementation strategies and the TMFs used to guide the development of the implementation strategies. A pilot test of data extraction was performed by Y.D. and L.G. on two studies using the Excel worksheet to ensure consistency. Final data extraction was completed independently by Y.D. and L.G. Any ambiguity identified was resolved through consensus discussion.

### 2.5 Data Analysis

The operational procedures for the implementation strategies were extracted from the included studies, categorized and then inductively coded (by Y.D.) to capture the strategy components. Coding was checked (by L.G.) and discrepancies were resolved by discussion. Subsequently, we used a directed content analysis approach, which systematically categorizes and evaluates data on an existing theory [24], to map the strategy components to the ERIC taxonomy. This mapping process was performed initially by Y.D., the results were then reviewed and discussed in the research team meetings until a consensus was reached. Further, to provide palliative care practitioners and researchers with specific insight into selecting implementation strategies, a meta theme encompassing the aspects of ‘what’, ‘who’ and ‘how’ was formed based on the identified implementation strategies. A descriptive summary of the characteristics of the included studies, the implementation strategies, the TMFs and the overarching meta themes were compiled and tabulated within evidence tables.

## 3 Results

### 3.1 Search Results

**Figure 1:**
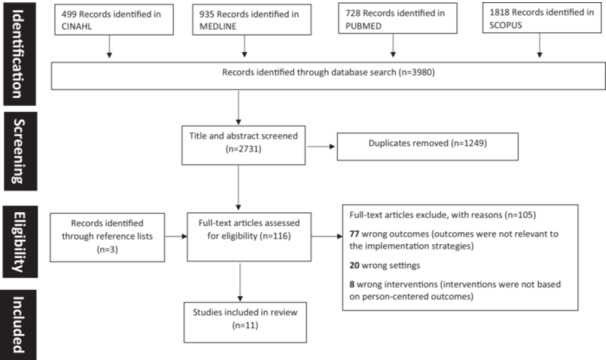
PRISMA flowchart of study selection and inclusion process.

A total of 3980 articles were identified, and 2731 articles remained for title and abstract screening after removing duplicates. One hundred and sixteen articles advanced to full‐text review, and 11 papers met the criteria for data extraction (Figure 1).

### 3.2 Study Characteristics

The characteristics of the included study are presented in Table 2. The 11 included studies originated from eight countries: The UK (*n* = 3) [14, 25, 26], Australia (*n* = 1) [31], USA (*n* = 1) [33], Canada (*n* = 1) [32], Italy (*n* = 1) [29], Brazil (*n* = 1) [28], Sweden (*n* = 1) [27] and Germany (*n* = 2) [30, 34]. All were published between 2017 and 2022. Four involved qualitative methods [14, 25, 26, 34], two used mixed methods [27, 28], one was a quantitative study [29], three were implementation studies [30, 32, 33] and one was a clinical update [31]. Eight studies were conducted in a single hospital‐based palliative care setting [25, 28, 29, 30, 31, 32, 33, 34] and the remainder were conducted in multiple settings [14, 26, 27]. All studies focused on improving the palliative care quality by improving clinical outcomes for inpatients, except for the Carer Support Needs Assessment Tool (CSNAT) intervention, which targeted support for family/carers [25].

**Table 2:** Characteristics of the included studies.

Study/year/country	Aims	Study setting	Study method	Data collection methods/Participants
A. Hall, 2020, UK [25]	To investigate the implementation of an evidence‐based Carer Support Needs Assessment Tool (CSNAT) intervention to support carers during hospital discharge at end of life	One National Health Service (NHS) Trust (hospital)	Longitudinal qualitative study	Data collection methods: Group and individual interviews, observation of regular team meetings, review of procedural documents
				Participants: A hospital‐based Supportive and Palliative Care Team and a Community Macmillan Team
J. Diffin, 2018, UK [26]	This study identifies how the PARIHS component of ‘facilitation’ and its interplay with the components of ‘context’ and ‘evidence’ affect implementation success	36 palliative/end‐of‐life care organizations	Qualitative study	Data collection methods: Qualitative repeat interviews, field notes
				Participants: A total of 38 internal facilitators (IFs): Clinical Nurse Specialist (CNS)—7 Social Worker—7 Head of overall service/management position (e.g., hospice at home team manager, family services manager)—16 Senior hospice at home team practitioner—2 Occupational therapist (OT)—2 Carer support lead/co‐coordinator—2 Other medical professional—2
S. Linder, 2018, Sweden [27]	To explore the feasibility of a pilot version of an implementation strategy for the Integrated Palliative Care Outcome Scale (IPOS) in acute care settings	3 acute care units (pulmonary diseases unit, neurological diseases unit and gastro‐surgery unit), and 1 palliative care unit	Qualitative and quantitative methods	Data collection methods: An interview guide with open‐ended questions, patient records
				Participants: (22 in total) 3 nurse managers and 2 internal facilitators (nurses), nurses and assistant nurses (2 staff members at the neurological unit, 4 at the pulmonary unit, 4 at the surgical unit and 6 at the palliative unit) and 1 physician at the palliative unit
A. P. Tavares, 2017, Brazil [28]	To implement a patient‐centred outcome measure in daily practice and fulfil one quality indicator: improve pain during the 72 h after admission, in at least 75% of patients	A specialist PC inpatient unit	Mixed study	Data collection methods: Qualitative data were gathered from notes taken consistently at each team meeting and whenever healthcare participants felt it was important to write thoughts/observations to discuss
S. Tanzi, 2020, Italy [29]	This study was aimed at developing, piloting and preliminarily assessing a complex intervention on pain management in hospitalized cancer patients	A specialized palliative care service (PCS) of an Italian hospital	Quantitative study: retrospective	Data collection methods: Data were collected from different pre‐existing electronic databases; data on patients' pain scores and PCS calls were retrospectively collected
C. Pinto, 2018, UK [14]	To explore how patient‐centred outcome measures are used in specialist palliative care, and identify key considerations for implementation	Nine specialist palliative care services in South London (UK), including one in‐patient hospice, five hospital and three community teams	Qualitative study	Data collection methods: Semi‐structured interviews and nonparticipant observation (field notes)
				Participants: ThirtyThirty‐eight‐eight participants were interviewed (39 approached): 7 patients, 4 family caregivers, 11 doctors, 8 nurses and 8 allied health professionals; Nine observations with 6 nurses and 3 doctors were undertaken (3 in the community, 5 in a hospital and 1 in an in‐patient hospice)
C. Bausewein, 2018, Germany [30]	To describe the process of implementing routine outcome measurement into daily clinical work in a university palliative care unit	A 10‐bedded palliative care unit in the Department of Palliative Medicine at Munich University Hospital	Implementation study	Data collection methods: Unclear
				Participants: Unclear
S. Aranha, 2018, Australia [31]	The Supportive and Palliative Care Unit (SPCU) responded by planning a quality improvement project which aimed to embed the Palliative Care Outcome Collaboration (PCOC) clinical assessments throughout the interdisciplinary team	The SPCU, Eastern Health	Clinical update	Data collection methods: Unclear
				Participants: Unclear
J. W. Neal, 2021, Canada [32]	To assess the feasibility of implementing a hybrid electronic and paper screening tool for distress in all patients coming to a large academic cancer centre and an associated integrated network site	An integrated academic cancer centre	Implementation study	Data collection methods: Questionnaires were completed before the visit on an electronic patient portal
				Participants: 26,242 patients
O. Generalova, 2021, USA [33]	We conducted a randomized clinical trial for the introduction of ePRO at an academic medical centre to (1) identify key features that could be applied in general clinical practice and (2) assess the impact on healthcare utilization	Stanford Cancer Centre	Implementation study	Data collection methods: Questionnaires were sent to patients and clinicians
				Participants: 72 patients and 32 clinicians
H. Seipp, 2022, Germany [34]	To explore how the Integrated Palliative Outcome Scale (IPOS), IPOS Views on Care (IPOS VoC) and the Short‐form Zarit Caregiver Burden Interview (ZBI‐7) can be feasibly, acceptably and appropriately implemented in the daily care routines of SOPC	Specialized outpatient palliative care	Qualitative study	Data collection methods: Focus groups with SOPC‐team members, field notes of meetings and conversations with the SOPC teams.
				Participants: Team members were mainly nurses and physicians (*n* = 14)

### 3.3 Identified Implementation Strategies

The number of implementation strategies used in each study ranged from 7 to 15, with a total of 115 unique strategies identified from the 11 included studies. These 115 strategies were mapped to 38 out of the 73 strategies across the nine categories of the ERIC framework. The most used ERIC categories were *train and education stakeholders* and *support clinicians*, followed by *develop stakeholder interrelationship* and *use evaluation and iterative strategies* (Supporting Information S1: Tables 2 and 3)*.*

### 3.4 Train and Educate Stakeholders Category

Twenty‐four implementation strategies were classified under this category in the ERIC framework. *Conducting educational meetings* was the most commonly used strategy and was employed in 8 out of the 11 included studies to introduce the QI program to the stakeholders [25, 27, 29, 30, 31, 32, 33, 34]. Education sessions were provided by either external facilitators or clinicians who were familiar with the program. Other frequently used strategies described were *conduct ongoing training* (*n* = 4) [14, 29, 31, 34], *make training dynamic* (*n* = 3) [14, 27, 30] and *distribute educational materials* (*n* = 3) [26, 27, 29]. The schedules for ongoing training varied, including a ‘2 years follow‐up phase’ [29], ‘regular training and education’ [14], ‘biannually’ [31]. To make the training more dynamic and flexible, training sessions considered the workload and the number of staff [27], a stepwise approach [14], a combination of different training methods such as role play, real‐time feedback and discussion and a weekend team retreat [30].

### 3.5 Support Clinicians Category

*Facilitate relay of clinical data to providers* was the most frequently used strategy within this category (*n* = 8 included studies) [14, 27, 28, 29, 30, 31, 32, 34]. The real‐time data obtained from the PCOMs were used at the multidisciplinary clinical handover, team meeting or communication with the patients and family/carers to support decision‐making, care planning or specialist referral. *Develop resource sharing agreements* was another commonly employed strategy within this category. Four out of 11 included studies highlighted support to clinicians from external facilitators to enable the implementation of the PCOM‐based QI program in routine clinical practice [26, 27, 31, 34].

### 3.6 Develop Stakeholder Interrelationships Category

*Develop stakeholder interrelationships* is the other most frequently used category of the ERIC framework (21 implementation strategies). *Identifying and prepare champions* (*n* = 4 included studies) [14, 25, 26, 27] and *involve executive boards* (*n* = 4 included studies) [26, 27, 31, 33] were the two most frequently employed strategies within this category. All four studies which employed the *Identifying and prepare champions* strategy emphasized that internal facilitators/champions should be health practitioners with clearly defined roles and responsibilities related to the intervention [14, 25, 26, 27]. Meanwhile, they should be fully supported by external facilitators [25, 26, 27], co‐internal facilitators [26] and executive boards of the hospital [26, 27].

### 3.7 Use Evaluative and Iterative Category

*Audit and provide feedback* was the most widely used strategy within this category and the most commonly used strategy in this review. Ten out of 11 studies employed this strategy [14, 25, 26, 27, 28, 29, 30, 31, 32, 34]. It included two types of feedback. The first was an ongoing reflection on the implementation process, including proactive and timely addressing of identified/potential problems, highlighting the importance of quantitative feedback, feedback forms and qualitative feedback [14, 25, 26, 27, 28, 29, 32]. The other type of feedback was periodically providing feedback from the patients' clinical outcomes to better implement the QI program [30, 31, 32]. *Stage implementation scale up* was used in four studies [14, 28, 29, 32], including a 6‐month pilot test before the formal implementation in two studies [28, 29].

### 3.8 Adapt and Tailor to Context Category

Within this category, *promote adaptability* was the most frequently used strategy (*n* = 6 included studies) [14, 25, 28, 30, 32, 33]. Five out of six studies emphasized that the PCOMs should be validated, easily used, specific to palliative care and practical for local palliative care services [14, 28, 30, 32, 33]. One study highlighted that the practitioners should have the freedom to fit the intervention into their daily practice while maintaining the intervention's fidelity [25].

### 3.9 Provide Interactive Assistance Category

*Provide local technical assistance* was a widely used strategy in this category (*n* = 6 included studies) [14, 26, 30, 32, 33, 34]. This strategy was represented by six studies in which the PCOMs were embedded into the electronic patients' medical records with the assistance of information technology (IT) staff. For ease of use in clinical practice, as well as for audit and service‐level use, one study highlighted that longitudinal and cohort PCOM data should be displayed by adapting the clinical IT system to fit the QI program into their daily practice while maintaining the fidelity of the intervention [14].

### 3.10 Change Infrastructure Category

In this category, *change record systems* was the most common strategy identified (*n* = 3 included studies) [26, 29, 30]. This strategy emphasized the importance of electronic devices in external audits [29] and integrating the PCOM assessment data into an electronic database [30]. One study also established a carer record embedded in the patient's electronic record to facilitate targeted support for family carers [26].

### 3.11 Utilize Financial Strategies Category

In this category, we identified two strategies from one included study: *alter incentive/allowance structures* and *fund and contract for clinical innovation* [14]. Policy and national drivers are the key aspects outside the organization influencing the use of PCOMs; importantly, the process of how to drive the policy changes based on the PCOMs data at both local and national levels should be transparent. Funds to support the uptake of PCOMs nationally were provided to facilitate the implementation in one study [14].

### 3.12 Engage Consumers Category

The sole strategy identified within this category involved *patients/consumers and family members*. Two studies employed this strategy, providing education to patients and/or nonprofessional caregivers on communicating their symptoms [29, 33].

#### 3.12.1 Meta Themes: Three‐Tiered Implementation Strategies

Three overarching themes from the perspectives of implementation science were synthesized from the identified strategies (Supporting Information S1: Table 4). This provides a summary of the strategies in three practical levels to promote the routine use of PCOM‐based QI programs in palliative care clinical practice: (a) What to do: build capacity, develop and share resources through training and education, establish stakeholder relationships and support palliative care professionals and integrate the PCOM‐based program into the current working procedure by changing infrastructure both at the organizational and cultural level. These were derived from the following categories of the ERIC categories: Train and educate stakeholders, develop stakeholder interrelationships, support clinicians and change infrastructure; (b) How to do it: be agile, responsive and interactive in the support provided (derived from ERIC categories: adapt and tailor to context, provide interactive assistance and utilize financial strategies); and (c) Who to do it with: involved all the relevant stakeholders, including palliative care professionals, patients, family/carers, those who provided education and implementation guidance, hospital administrative leaders/staff and those involved in funding services (derived from all ERIC categories).

#### 3.12.2 TMFs

Four studies described the TMFs selected for the design of the implementation strategies of their study (Supporting Information S1: Table 2). The following TMFs were used: the Promoting Action on Research Implementation in Health Services (PARIHS) Framework [26], the Consolidated Framework for Implementation Research (CFIR) [14], the MRC framework [29] and the PDSA (plan, do, study, act) continuous improvement methodology [31]. Only one study followed Proctor, Powell and McMillen's recommendations for the reporting of implementation strategies to clearly describe the components of their implementation strategy in detail [13, 26].

## 4 Discussion

This scoping review identifies the implementation strategies used and the application of the TMFs in designing the implementation strategies to promote the implementation of PCOM‐based QI programs in hospital‐based palliative care settings. We mapped the implementation strategies to the strategies and categories of the ERIC framework using uniform language to ensure clarity about the kinds of strategies that have been adopted in palliative care. We found that 52% (38/73) of the ERIC strategies had been used in the included studies. The majority of identified strategies (63%, 24/38) were located in the Go‐zone quadrant I of the ERIC compilation, meaning that both the importance and feasibility ratings are above the scale means [18].

Developing tailored implementation strategies based on the identified barriers, facilitators and contextual resources is regarded as a pre‐requisite for successfully implementing innovation into routine clinical practice [35, 36, 37, 38]. However, only two studies in our review detailed the process of developing the implementation strategies based on the identified barriers and facilitators [27, 29]. A previous study has indicated that a critical reason for the persistent lag between evidence and practice in healthcare services is the minimal effort made to identify factors that could facilitate the success of implementing evidence‐based interventions [39]. As a result, the majority of implementation strategies are based on best assumptions rather than on identified barriers and enablers [40, 41]. Our study also suggests that many implementation strategies were not developed based on identified barriers and facilitators. To better implement PCOM‐based QI programs in palliative care, future research and practice should focus on developing tailored implementation strategies through the thorough identification of barriers and facilitators.

It is critical to design implementation strategies according to implementation TMFs to provide a clear understanding of how and why specific implementation strategies lead to specific implementation outcomes [42]. The use of TMFs to guide implementation can also optimize the generalization of the findings and contribute to the future reproducibility of implementation strategies. Despite the recognized importance of using TMFs, only four studies included in this review employed TMFs to guide the design of the implementation strategies. A similar situation exists in other healthcare settings. For example, a scoping review in the field of occupational therapy for adult stroke rehabilitation reported that less than half of the included studies (12 out of 26) applied implementation TMFs to develop strategies [36]. With the increasing number of TMFs discussed in the implementation literature, selecting the appropriate TMFs is essential. The Dissemination & Implementation Models Webtool [43] and the Theory, Model and Framework Comparison and Selection Tool (T‐CaST) [44] are designed to assist researchers and practitioners select an appropriate TMF for their project.

Importantly, even though we synthesized a three‐practical level approach (what to do, how to do it and who to do it with) to facilitate the routine use of the PCOM‐based QI program, implementation remains a complex, dynamic process. This process involves ongoing changes in staff cognition, attitudes and emotions. Therefore, it is crucial to clearly address the three questions—what to do, how to do it and who to do it with—at the three different implementation phases: pre‐implementation, implementation and sustainability [45]. Additionally, to further refine the ERIC framework, implementation strategies could be systematically categorized according to these implementation phases [17].

One noteworthy difficulty encountered during this review was identifying/extracting the implementation strategies used in the studies from each publication. Implementation strategies were not reported in a standardized manner and were often poorly described. Furthermore, the components of the implementation strategies were fragmented and scattered across different sections of the publications. Only one study specified the implementation strategies using Proctor's recommendations [13], clearly reporting the actors, actions, action targets, temporality, dose, affected implementation outcomes and justification [26]. Without a common language to define and describe the components of the implementation strategies, researchers and practitioners will find it challenging to replicate these implementation strategies or select the optimal combination of implementation strategies for future study or practice. A potential consequence of this may be the emergence of a ‘secondary’ research‐to‐practice gap [46] because the empirical findings and knowledge from the field of implementation science cannot be advanced into clinical practice. Therefore, we recommend that implementation strategies be described in sufficient detail. Key components, such as the detailed description of the strategy, contextual factors, stakeholder involvement, resources and materials used, implementation phases and timeline, training and support provided and adaptation mechanisms should be reported transparently and labelled consistently to enable replication and measurement.

## 5 Limitations

There were limitations to this study. First, we did not perform a quality appraisal assessment for the included studies as the purpose of this scoping review was to provide a ‘map’ of the existing evidence, regardless of the methodological quality or the risk bias of the study. The current PRISMA‐ScR reporting guidelines support our approach by stating that critical appraisal is not mandatory for scoping reviews depending on the aims of the review [47]. Second, we conducted a systematic search to capture all possibly relevant studies with the assistance of a university librarian. However, only 11 studies met the inclusion criteria, which may suggest the under‐utilization of implementation strategies to integrate the PCOMs in palliative care or a search strategy that was too restrictive (however, we think this is unlikely given our knowledge of the literature and input from experts allied to the project). To help address this, future research should focus on employing implementation science and methodology to accelerate the uptake of PCOM‐based QI programs in palliative care settings. Lastly, given that the descriptions of implementation strategies were fragmented and scattered across different sections in the majority of included publications, there was a risk that we may not have comprehensively extracted all the strategies used. To mitigate this risk, two researchers extracted the information independently, and any discrepancies were resolved through discussion.

## 6 Conclusions

Implementing PCOM‐based QI programs in hospital‐based palliative care is an ongoing, interactive and dynamic process. Our findings suggest that future implementation projects should focus more on developing tailored strategies based on the identified/potential barriers, facilitators and contextual resources at the different implementation stages. To generalize the findings by providing common language and constructs that enable consistently articulated explanations of implementation‐related phenomena, we also recommended the use of rigorous implementation TMFs to guide the development of implementation strategies. In addition, in future studies on promoting the uptake of PCOM‐based QI programs, the components of the implementation strategies should be reported transparently and labelled consistently to enable replication and measurement, preferably using a standardized reporting guideline.

## Author Contributions

**Yunyun Dai:** conceptualization, methodology, writing–original draft, writing–review and editing, project administration, resources, funding acquisition, validation, data curation, formal analysis, visualization. **Barbara A. Daveson:** conceptualization, methodology, validation, funding acquisition, resources, writing–review and editing, visualization, supervision, project administration. **Luyu Gan:** formal analysis, data curation, writing–review and editing, validation. **Jinfeng Ding:** conceptualization, methodology, resources, funding acquisition, supervision, project administration, writing–review and editing, data curation, validation, visualization, formal analysis. **Yongyi Chen:** conceptualization, methodology, writing–review and editing, supervision. **Claire E. Johnson:** conceptualization, methodology, validation, data curation, formal analysis, supervision, resources, project administration, writing–review and editing, visualization.

## Ethics Statement

Ethical approval was not required for this scoping review as data used for analysis were extracted from published studies.

## Conflicts of Interest

The authors declare no conflicts of interest.

## Supporting information

Supporting information.

## Data Availability

Data sharing is not applicable to this review as no new data were created or analysed in this study.
