# Supplementary material for: Implementation Strategies for Quality Improvement in Palliative Care: A Scoping Review
Source: Health Expect. 2024 Jul 26;27(4):e14151. doi: 10.1111/hex.14151 (PMC11273214; doi:10.1111/hex.14151)
Supplement: Supplementary file 1 — Supporting information. [file HEX-27-e14151-s001.docx]

**Supplementary table 1 Search strategy used in Medline**

| #1 SU Palliative care or hospice and palliative nursing or palliative medicine or palliative support or end of life or supportive care or terminal care or hospice care (86121) |
| --- |
| #2 AB patient-centred outcome* or patient-reported outcome* or outcome assessment* or person-centred outcome* or PRO or PROMs or PROMIS or proxy-rated outcome* or clinician-rated outcome* or proxy-reported outcome* or carer-reported outcome or care-rated outcome or clinician-reported outcome* or person-centred intervention (457,575) |
| #3 Implement* or sustain* or integrat* or embed* (1,995,199) |
| #1 and #2 and #3 |
| Refine by: Language: English; date of publication limited from January 1^st^ 1990 to 12^th^ October 2022 (772) |

Note: SU=Subjects, AB=Abstract

**Supplementary table 2 Identified implementation strategies and the applied TMFs**

| **Study/year/Country** | **QI programs based on PCOMs** | | | | **Implementation strategies** | | **Implementation strategies guided by TMFs** |
| --- | --- | --- | --- | --- | --- | --- | --- |
|  | **Name** | **Tools** | **Rater** | **Assessment frequency** | **Strategy Components** | **Operational procedures** |  |
| Hall A., 2020, UK[25] | Carer Support Needs Assessment  Tool (CSNAT) intervention: Two components of the intervention: the ‘tool’ and the ‘approach’ | Carer Support Needs Assessment  Tool | Clinician or carer | Before hospital discharge | 1.Training workshops | - Participants took part in two face-to-face training workshops - Trained the full teams in how to use the intervention | N/A |
|  |  |  |  |  | 2.A service-level implementation strategy | - A core group who would lead implementation developed the implementation strategies |  |
|  |  |  |  |  | 3.Identity a champion | - A hospital team nurse - Roles: Development and communication of the implementation strategy and regular updates at team meetings |  |
|  |  |  |  |  | 4. Provide project support from leadership | - An administrator was given dedicated time to provide project support. |  |
|  |  |  |  |  | 5. Adjust the intervention more flexible | - Practitioners had freedom to fit the intervention into their daily practice (while maintaining its fidelity) |  |
|  |  |  |  |  | 6.Reminder system | - Keep the intervention visible in daily routines - Regular project-specific meetings - Incorporating project updates in team briefs |  |
|  |  |  |  |  | 7.Ongoing feedback | - Practitioners were encouraged to have ongoing reflection on the implementation process. - Developed feedback forms for themselves to capture experiences of intervention use - Quantitative feedback - Highlighted the importance of discursive reflection |  |
| Diffin, J., 2018, UK[26] | Carer Support Needs Assessment  Tool (CSNAT) intervention | Carer Support Needs Assessment  Tool | Clinician or carer | Daily | 1.Evidence   - Legitimising the intervention to change practice - Informed advocacy: ability to distinguish intervention from existing practice | - One lead IF initially did a presentation to the team about the importance of assessing and addressing carers needs which appeared to provide the legitimacy for introducing a person-centred process of assessment and support for family carers - The lead IF demonstrated a fuller understanding of the intervention and the importance of following each stage, and distinguished the use of the CSNAT intervention from previous practice | The Promoting Action on Research Implementation in  Health Services (PARIHS) Framework |
|  |  |  |  |  | 2. Context   - Leadership support - Organisation culture - Establish a carer record with IT staff | - An ‘organisation’ agreement is signed by senior management to indicate they agree with providing the resources for the IFs to fulfil their role, including time - Dedicate to carers in the same way with the patients - IFs had to consult with Information Technology (IT) to create a carer record on the system |  |
|  |  |  |  |  | 3. External facilitators-Members of the CSNAT team | - The CSNAT team who act as external facilitators (EFs). - EFs support IFs with the following activities:   - Reflection on their organisation’s ethos or mission statement (often highlights they are there for the carers/family/friends of the patient)  - Considering how they currently became aware of carer support needs  - Planning for how they could use the CSNAT intervention in their individual practice  - Making an initial ‘implementation plan’ for their service to include thinking about how to use the intervention within the service, where to record data on carers, format of CSNAT documentation, and how they could deliver training to and support their colleagues |  |
|  |  |  |  |  | 4.Development and distribution of the ‘CSNAT training’ toolkit | - Development of the toolkit was based on previous experience with services and on feedback from practitioners who have used the intervention - All IFs provided with a ‘CSNAT training’ toolkit which includes materials covered at the training day and hints and tips on how to implement the CSNAT intervention in practice, both at individual and organisational level - A power-point presentation and accompanying notes are also supplied if IFs want to make use of this in the training sessions they host for their colleagues |  |
|  |  |  |  |  | 5.Trained the IFs | - All IFs attend a ‘CSNAT training day’ hosted by the CSNAT team who act as external facilitators (EFs). Training delivered on the CSNAT intervention evidence base, and a detailed overview of how to use in practice (including case study examples from other practitioners) |  |
|  |  |  |  |  | 6.Continuous consultation and peer support | - All lead IFs are asked to participate in monthly one-hour teleconferences with the CSNAT team (EFs) and lead IFs from other sites for the purposes of peer support and shared learning on implementing the CSNAT intervention at an organisational level. Email and telephone support also available from EFs |  |
|  |  |  |  |  | 7.Identify and clarify the roles of the Internal facilitator | - Each service within an organisation that is implementing the CSNAT intervention selects 2–3 practitioners to be internal facilitators (IFs); referred to within each site as ‘CSNAT Champions’. One practitioner is asked to take on the role of the ‘lead’ IF - The lead or co-IFs had the authority within the team to advocate a change in practice and make adjustments to support the implementation - The lead IF was supported by co-IFs - The IFs cascaded this training to colleagues and supported them in using the CSNAT intervention - Roles: (i)Authority to make change, (ii)Support from co-facilitators, (iii)Reach of IF in service: ability to deliver training & support |  |
|  |  |  |  |  | 8.Internal facilitation process:  (i)Collaborative approach: bring others on board  (ii)Continuous communication  (iii)Proactive problem solving | - Engaging additional staff as IFs - Supporting their colleagues in the use of the CSNAT intervention - Holding regular discussions with colleagues on issues related to using the CSNAT intervention in practice (both at formal meetings and during informal exchanges) - IFs who communicated effectively about the intervention, regularly reminded their colleagues about its use and addressed their fears, worries or anxieties about using it in their practice - IFs who regularly reflected on the progress of the implementation, identified potential problems and then made changes to address these |  |
| Linder S., 2018, Sweden[32] | The Integrated Palliative care Outcome Scale (IPOS) | Two versions of IPOS, one for self-reporting by the patient and one for proxy-estimation by healthcare professionals | The patient or the proxy | IPOS was to be used on the third day of care at the unit. If the patient was still in care at the seventh day after admission, health care professionals were to perform a follow-up using IPOS | 1. Identify barriers and opportunities, and tailor the implementation strategy to the context | - An interview study was conducted to identify obstacles and opportunities for implementation. The findings contributed to the design of the strategy | N/A |
|  |  |  |  |  | 2. External facilitators | - Nurse manager and the IF participate in a meeting with external facilitator before the start of the study - The assignments of the nurse managers and internal facilitators were to be discussed in meetings with the external facilitator - The nurse manager is in turn supported by the external facilitator at the planned visits to the unit according to the project plan - The internal facilitator is supported by the nurse manager as well as the external facilitator at the planned visits/contacts to the unit according to the project plan |  |
|  |  |  |  |  | 3. Information meetings with external facilitators, an information leaflet was distributed | - Week 1: The information meeting introduced the project and an information leaflet was distributed, the meetings lasted between 10 and 20 minutes |  |
|  |  |  |  |  | 4. Identify internal facilitators | - Nurse manager appoint 1-2 nurses to function as internal facilitator in the study |  |
|  |  |  |  |  | 5.Leadership support | - Nurse manager support the appointed nurse(s) in her/his assignment as internal facilitators - Nurse manager support staff to identify patients to be offered to complete IPOS |  |
|  |  |  |  |  | 6. Internal facilitators roles | - IF participates in a meeting with the nurse manager and external facilitator before the start of the study - IF Supports staff to identify patients to be offered to complete IPOS - IF support staff to offer patients to complete IPOS - IF supports staff to use proxy version of IPOS when necessary - IF supports staff to initiate actions in cooperation with the patient, next of kin and other professionals |  |
|  |  |  |  |  | 7. Use the assessment results | - IF Supports staff to discuss the patient’s scorings in IPOS with the patient, next of kin and at team meetings when necessary |  |
|  |  |  |  |  | 8. Provide feedback | - IF give feedback to staff related to the use of IPOS. - Act as a contact between the staff and the external facilitator regarding questions and uncertainties related to the use of IPOS. |  |
|  |  |  |  |  | 9. Training sessions regarding IPOS (flexible) | - Training session allowing the units to set their own schedules based on current workload and number of employees. - Discussions and reflections were encouraged during the sessions and participants received PowerPoint handouts |  |
|  |  |  |  |  | 10.IPOS clinical guideline was distributed | - After the training period, the basic and the pocket versions of the clinical guidelines were distributed to the units |  |
|  |  |  |  |  | 11.Conduct ongoing consultation | - The training was to be followed by 12 weeks of clinical use of IPOS - The external facilitator’s visits to the units were scheduled in the project plan   -Week 1: 3–4 visits  -Weeks 2–4: visits twice a week  -Weeks 5–8: visits once a week  -Weeks 9–12: telephone or e-mail support once a week |  |
| Tavares AP., 2017, Brazil[31] | The Palliative care Outcome Scale (POS) | The Brazilian version of the Palliative care Outcome Scale (POS-Br) and POS-S (Palliative care Outcome Scale-Symptoms) | Patients or the proxy (a family  member, carer or nurse) | POS and POS-S were filled by all patients on admission  (T0), between the third and fifth days (T1) and once a week  after that | 1. Trained the unit director to be the trainer in the service | - The unit director (APST) attended a training workshop which included clinical and research components | N/A |
|  |  |  |  |  | 2. Choosing PCOMs suitable for the service | - Specifically developed for palliative care patients - Validated in Portuguese - Easy and practical to use - Freely available |  |
|  |  |  |  |  | 3. Pilot test before the formal implementation | - A 6-month pilot implementation was conducted |  |
|  |  |  |  |  | 4. Model for pilot study and modified during the post-pilot period | - A model for pilot study was conducted - New rules established during the post pilot period - POS-S was modified - to conduct the main study - Understanding and discussing difficulties during the pilot was crucial to develop new processes for both measures in daily practice |  |
|  |  |  |  |  | 5.Understanding and discussing difficulties via meeting   - Importance of using a measure routinely - Defining who applies the measure to which patients - Empowering the PC team to use POS and Modified POS-S results - Using results in real time | - Discussions (n = 5) took place looking at changes to the processes - All members of the palliative care team should understand the real benefits of using a questionnaire, for patients, relatives, the institution and the PC team itself - The palliative care team should clearly define who will use the questionnaire, in which patients and with which periodicity. The team should also consider and decide on quality indicators that will be monitored when making these decisions - The palliative care team needs to take into account POS and Modified POS-S results within daily practice to understand patient needs and establish a therapeutic plan and goals of treatment - The palliative care team should use POS and Modified POS-S results to improve the care provided |  |
| Tanzi S., 2020, Italy[30] | A quality  improvement program on cancer pain  management | The UK  Medical Research Council (MRC) | The patient or the proxy | Daily | 1. Analysing the local context (professionals and patients) | - The observation period in the ward by an expert allowed us to understand the knowledge of nurses and physicians about pain evaluation and treatment - General knowledge about pain, its evaluation, and treatment of the professionals were assessed by the self-perceived knowledge questionnaires - These results permit the PC physician implementing the intervention to tailor both frontal lessons and bedside training to professional teaching needs | The UK Medical Research Council (MRC) |
|  |  |  |  |  | 2. Modelling the intervention | - A 5-component intervention was designed, taking into account evidence from the literature and the specificity of the context |  |
|  |  |  |  |  | 3. Assessing the quality of the intervention by setting indicators | - Set of indicators and their rationale to quantify the extent to which the component was achieved |  |
|  |  |  |  |  | 4. Pilot implementation of the intervention | - This quality improvement project includes a pilot phase designed as a phase 0–I study (according to the MRC framework) and a long follow-up phase (2 years) over the program |  |
|  |  |  |  |  | 5.Ward professional  training | - Both physicians and nursing staff are trained in managing patients’ pain appropriately |  |
|  |  |  |  |  | 6. Education for both patients and nonprofessional caregivers | - The admitted patients were provided with the leaflet on pain |  |
|  |  |  |  |  | 7. Distributed leaflet to the patients | - Patients receiving the leaflet during inward admission |  |
|  |  |  |  |  | 8. Daily pain assessment and specialist  referral in time | - Average 24-hour pain was evaluated by the nursing staff for all patients able to answer - The discussion on pain scores occurs daily - All patients with 2 days of severe pain were referred to the PCT |  |
|  |  |  |  |  | 9. External  Audit | - Data on patient pain management in the ward are periodically monitored by the hospital management and discussed - Hospital management perform an external audit every 6 months |  |
|  |  |  |  |  | 10. Follow-up phase   - An electronic device was used - Ongoing training for clinicians | - Insert the indicator “leaflet delivery” exactly at the bedside by the use of an electronic device - Complete the training course for all physician and nurses |  |
| Pinto C., 2018, UK[15] | The Outcome Assessment and Complexity Collaboration  (OACC) project | Phase of illness, modified Australian Karnofsky Performance Status (AKPS) ,  Integrated Palliative care Outcomes Scale (IPOS) | The patient or the proxy | Daily | 1. Intervention   - Appropriateness and validity of PCOMs in palliative care - Pilot test | - Careful selection of validated PCOMs that are palliative care specific and useful for palliative care clinical teams - Clearly present the advantages of using PCOMs to professionals, particularly those emphasized by patients and family caregivers - Pilot the outcome measures and address any stakeholders’ concerns, e.g., provide staff training, allow for protected time to complete the outcome measures, demonstrate how data from outcome measures will be used | The Consolidated Framework for Implementation Research (CFIR) |
|  |  |  |  |  | 2. Inner setting   - IT infrastructure - Healthcare environment - Team working - Utilisation in daily interactions - Support leadership | - Creation of interface in IT systems that support collection of PCOMs remotely to avoid duplication of effort - Adapt IT systems to display: (I) longitudinal PCOMs, for ease of use in clinical practice; and (II) enable reporting of cohort PCOMs data for audit and service-level use - PCOMs need to be identified as important and integral to the care provided by using them to support decision making at clinical and strategic levels - Early involvement of the entire team in the training and troubleshooting during the implementation process |  |
|  |  |  |  |  | 3. Outer setting   - Policy driver - Funding | - Policy drivers are powerful incentives for change. Policy makers need to be transparent about what data is expected from services and how PCOMs data will be used to inform policy at both local and national levels - Provision of funding, at least partial, to support uptake of PCOMs nationally |  |
|  |  |  |  |  | 4. Individual:   - Knowledge and competence - Attitudes towards PCOMs | - Introduce staff to the PCOMs, explain rationale for using them and provide training in the relevant communication skills - Address any concerns and misconceptions about PCOMs - Regular training and education are needed to ensure PCOMs are used consistently and reduce any subjectivity |  |
|  |  |  |  |  | 5. Implementation   - Stepwise introduce - Feedback - Champions & facilitators | - Ensure clarity about PCOMs to be introduced, when and how this will be done - Provide regular feedback sessions to engage and motivate staff   Identify champions and facilitators to support and encourage PCOM use |  |
| Bausewein C., 2018, Germany[33] | The Outcome Assessment and Complexity Collaborative  (OACC) | The Integrated Palliative Care Outcome Scale  (IPOS),  Phase of illness,  The Australian Karnofsky Performance Status  (AKPS) | Patients or the proxy | Daily | 1.Selection of outcome measure(s) | - The head of department and the clinical leads agreed to implement outcome measurement into the daily clinical routine of the palliative care unit - The focus of outcome measurement and respective tools should be on most prevalent symptoms of patients - Burden of relatives should also be part of the assessment - Internationally comparable outcome measures were chosen for routine clinical use - Measures had to have demonstrated reliability and validity for a palliative care population as well as responsiveness to change | N/A |
|  |  |  |  |  | 2. Education about the measure and use of results | - The team was introduced to outcome measurement in several team meetings including introduction of outcome measurement in palliative care in general, rationale and explanation of the chosen measures and role plays on how to use the measures - Education sessions were provided by members of the clinical and research team with specific experience in outcome measurement and also in providing training for outcome measurement in palliative care - To deepen the understanding and experience, a weekend team retreat focussed on outcome measurement in palliative care - Team members learned to use outcome measures, especially IPOS, in a 15-minute role play encounter between a professional and a patient with one observer, with time for feedback to the “professional” and discussion afterwards. In a second role play, a team meeting was simulated with 4–6 professionals discussing a patient’s problems of the last days and measures for management based on IPOS, AKPS and phase of illness - For IPOS scores 0–1: checking with patient whether and what support is necessary; for IPOS scores 2, 3 or 4 indicating a more severe problem or symptom burden, checking the management plan and discussing it in the multi-professional team. |  |
|  |  |  |  |  | 3. Selection of one coordinator/facilitator | - The consultant responsible for the palliative care unit was chosen as coordinator for outcome measurement on the ward. The team members, who also provided the training sessions served as facilitators on the ward. |  |
|  |  |  |  |  | 4. Who applies the measure and its periodicity | - On admission to the palliative care unit, a baseline assessment is completed by the responsible physician for every patient including phase of illness, IPOS and AKPS |  |
|  |  |  |  |  | 5. Records integrated into the electronic databases | - Questionnaires should be included in the patient record or entered in the electronical database - An electronic patient record was introduced in the Department of Palliative Medicine |  |
|  |  |  |  |  | 6. Analyses of outcome measures over time | - To illustrate the change in symptom scores over time, e.g., in relation to phase of illness, data can be presented in form of spider maps to show the team effects of care but also potential areas for improvement. |  |
| Aranha S., 2018, Australia[27] | The Palliative Care Outcomes  Collaboration (PCOC) | The Palliative Care Phase (PC  Phase), the Palliative Care Problem Severity  Score (PCPSS), the Symptom Assessment Scale  (SAS), the Resource Utilisation Group  Activities Daily Living (RUG-ADL), the Australian Karnofsky  Performance Scale (AKPS) | Patient or the proxy | Daily | 1. Improving the quality of the implementation through data entry and data review | - Data entry was reallocated to administration roles instead of clinical team members. Education was provided to administrative staff to ensure consistent practices in data entry. Real-time data reviews by nursing management were introduced to ensure clinical assessments were in keeping with the patient symptoms and phase of care. | 1.PDSA (plan, do, study, act) continuous improvement methodology  2.‘A3’ process improvement tools.  3.Eastern Health’s improvement methodology 4. performance excellence systems.  5.The Model of Care project’s |
|  |  |  |  |  | 2. Ongoing training | - A full PCOC training day for nursing staff and biannual PCOC training requirements being incorporated into position descriptions to emphasise the expectation to regularly undertake refresher training |  |
|  |  |  |  |  | 3. Education offered by the PCOC team | - All other staff were offered the opportunity to attend a PCOC training day, as well as shorter in-services within the SPCU run by the collaborative quality improvement team |  |
|  |  |  |  |  | 4. Team meetings | - Team meetings were changed to bi-weekly, all patients were discussed, using a team meeting tool that had been implemented prior to the unit undertaking PCOC assessments. The new regimen was pilot-tested and revised (PDSA method). - The newly designed team meeting tool incorporated reviews of patients’ function and symptoms as assessed by the PCOC tools and assisted in decisions to refer to allied health |  |
|  |  |  |  |  | 5. Use the assessment result | - PCOC assessments were further embedded at the daily inter-disciplinary clinical handover where informal care planning was undertaken - The PCOC assessment tools were augmented to provide suggestions for clinician interventions and an escalation pathway to medical (including after hours) and allied health staff when primary interventions did not improve symptoms |  |
|  |  |  |  |  | 6. Support from a restructured consultant | - The consultant roster was restructured so that there was a senior medical presence six days each week to support the recognition of and responsiveness to patients with complex or uncontrolled symptoms |  |
|  |  |  |  |  | 7. Small scale changes | - The SPCU leadership team identified the following areas for improvement and implemented changes in a stepped process over a period of months - The implementation of changes were discussed weekly by the SPCU leadership team and at the Model of Care project meetings by the wider inter-disciplinary team |  |
|  |  |  |  |  | 8. Audit and feedback | - Patient outcomes were reviewed six-monthly with the receipt of a consolidated report from the PCOC program |  |
| J.W. Neal, 2021, Canada | An electronic or on-paper modified Patient-Reported Outcomes Measurement Information System-Global Health questionnaire | PROMIS Global Health (GH v1.2) | Patient or the proxy | (1) Ahead of the visit to be completed before check-in, via a secure patient portal, or (2) in clinic on paper after check-in of the visit. | 1. Preparation for implementation   - Establishment of a Screening Committee - Selection and modification of PCOMS | - Key leadership formed a distress screening committee - Committee members included members from clinical operations, psychosocial care (social work, palliative care, hospital chaplain, and survivorship), quality leadership, and clinical teams (physicians [MDs], nursing [RNs], and advanced practice providers [APPs]) - Selected the validated and appropriate instruments for emotional distress - The questionnaire was modified in two ways | N/A |
|  |  |  |  |  | 2.Intervention developed based on the assessment | - An interdisciplinary group met weekly to design the initial intervention |  |
|  |  |  |  |  | 3.Pilot test before the formal implementation | - Lead-In Period: The initial PROMIS tool was piloted as a paper questionnaire to assess the feasibility |  |
|  |  |  |  |  | 4.Training | - Training included workshops with the medical assistants (MAs), RNs, MDs, and APPs - A tip sheet was developed for staff to assist with difficult conversations - The pilot launch was coordinated with an EHR training update |  |
|  |  |  |  |  | 1. Electronic Health Record (EHR) Integration | - The PROMIS tool was converted to an electronic questionnaire using existing EHR functionality - Three days in advance of a scheduled follow-up appointment, questionnaires were made available to patients via the patient portal. |  |
|  |  |  |  |  | 1. The clinical workflow development  - Reminder system - Assessment handling - Integration the supportive services for patient care | - Inviting patients to complete the questionnaire before the appointment. Patients received either an alert on a mobile device or an e-mail asking them to complete the questionnaire - A notification at check-in reminded the staff to give patients a paper copy of the questionnaire if they had not done so before the visit - the MAs to record the results of the on-paper questionnaire in the EHR or assist the patient to fill out the screening questionnaire on the computer in the examination room - The MA would also verbally alert the clinical team if the questionnaire resulted in a positive screen, with the expectation for the clinical team (APP and MD) to address the response as needed. Of note, positive screen answers were also visible on the main view of the EHR - To appropriately handle the anticipated need for supportive services, the cancer center supported the development of an umbrella organization (Cancer Care Services) that included supportive services including social work, palliative medicine, survivorship, nutrition, and rehabilitation therapy |  |
|  |  |  |  |  | 1. IT continuous support | - Throughout implementation, information technology teams provided continuous support to identify process issues and generated formal audit reports showing completion rates and other relevant metrics |  |
|  |  |  |  |  | 1. Refine the workflow through iterative feedback | - We then continued to assess this workflow in an iterative feedback process based on frequency of questionnaires and collected data through the first year of implementation |  |
| O. Generalova, 2021, USA | A cloud-based electronic  patient-reported outcome (ePRO) platform | symptom questionnaires (SQs) |  |  | 1. Per-implementation  - ePRO selection - EHR integration and data security - Stakeholder and leadership engagement - Clinical workflow development | - The Noona platform is a cloud-based mobile service, designed to capture PROs in oncology - The platform met requirements for future integration into our EHR platform, full integration was not implemented for the study period - Buy-in prior to the ePRO launch was prioritized. In 2015–2016, we obtained input and approval from the three oncology disease groups, the Information Technology department, the chief technical officer, and the director of operations - The workflow involved patients filling out the SQ on the ePRO platform, and the CRC reviewing those symptoms and uploading the document to the EHR as a clinical note. A PDF file of the SQ also showed a calendar view and printout of the patient’s symptoms for providers for easy reference. The Clinical Research Coordinators (CRCs) also provided a verbal summary to the clinician prior to the visit. Te EHR portal was still used for messaging for patients given its routine use in our clinical practice | N/A |
|  |  |  |  |  | 1. Implementation  - Training for the research team - Clinical care team training and engagement - Patient onboarding - Reminder system | - Bi-weekly study meetings of the research team with platform staff promoted a collaborative approach and facilitated troubleshooting - CRCs trained their respective clinical care teams to explain the eligibility criteria and intervention. The thoracic, gastrointestinal, and genitourinary clinical groups were enrolled consecutively, rather than simultaneously, allowing for individualized problem-solving to accommodate each clinic’s unique workflows - The CRC educated patients on how to communicate symptoms with their clinical teams via e-secure messaging, phone, and/or the ePRO - Patients received prompts to complete a SQ at least every three weeks or up to two days prior to upcoming oncology clinic visits. If the patient failed to complete the SQ, the CRC sent a reminder via the EHR portal |  |
| H. Seipp, 2022, Germany | Specialised outpatient palliative care (SOPC) | Integrated Palliative care Outcome  Scale (IPOS), IPOS  Views on Care (IPOS VoC), and Short-form Zarit Caregiver  Burden Interview (ZBI-7) | IPOS: Patient self-reporting or proxy-reporting by relatives or staf;  IPOS VoC: Patient self-report;  ZBI-7: Relative self-report | IPOS, IPOS VoC: On admission, after about 5–10 days,  and at≥3 further appointments; at  least once during further care, and  when changes occurred;  ZBI-7: On admission; at least once during  further care | 1. Initiation: preparation and team training | - We explained the diferent measures to the teams, and talked about content, aims, how to use them with the respondents in practice and benefits of the outcome measures in practical care - We gave the teams case folders containing information on the measures - We started using the paper-based version in order to be able to adjust the case folder flexibly, and to allow for written comments | N/A |
|  |  |  |  |  | 1. External support | - We encouraged the SOPC-team members to contact the study team in case of questions, comments, and problems arising from the use of the measures. Evaluation meetings were arranged when there was a need to discuss matters face-to-face - We wrote field notes on all telephone calls and meetings for preparation, training and evaluation. Field notes were taken of the date, team, type of contact, content, and the researchers’ comments |  |
|  |  |  |  |  | 1. Continuous feedback and adjustment | - To examine integration into daily care, we applied an iterative process, which included testing their use in SOPC, collecting feedback from health professionals, adjusting the process depending on the feedback, and examining the effect of the adjustments |  |
|  |  |  |  |  | 1. IT support | - Four teams started by using the paper version and switched to the software version later. The fifth team started using the software version directly |  |
|  |  |  |  |  | 1. Continuous training | - We therefore revised the training to focus more on usage and usefulness |  |
|  |  |  |  |  | 1. Understanding the usefulness of the PCOMs to confirm further buy-in | - One topic in these meetings was the potential for improvement that the outcome measures offered in individual cases, and their usefulness in daily practice, for example in visualising care and communication processes in a team - We further discussed the importance of measuring quality in the healthcare system, and spoke about the weight attached to data sovereignty to reduce fears of misuse. - The use of aggregated data to achieve internal quality improvements and to help explain quality of care to external audiences were further topics of the meeting |  |
|  |  |  |  |  | 1. Enable sensitive use | - To ensure the survey is used with respect for sensitive topics, we encourage SOPC-team members to integrate the patients’ self-reported views into conversations, to abstain from using the question’s exact wording if necessary, thus relying more on the patient’s narrative |  |

**Supplementary table 3 Identified implementation strategies mapped to the ERIC strategies and categories**

| **Categories in ERIC** | **Strategy in ERIC** | **Strategy components in the included studies** | **Number of studies** |
| --- | --- | --- | --- |
| **Train and educate stakeholders** | Conduct ongoing training (I) | - Ongoing training for clinicians[30] - Individual _ Knowledge and competence and attitudes towards PCOMs (Regular training and education)[15] - Ongoing training[27] - Continuous training[34] | 4 |
|  | Conduct educational meetings (I) | - Training workshops[25] - Ward professional training[30] - Education about the measure and use of results[33] - Education offered by the PCOC team[27] - Information meetings with external facilitators, an information leaflet was distributed[32] - Training[29] - Training for the research team and clinical care team training and engagement[28] - Preparation and team training to understand the usefulness of the PCOMs to confirm further buy-in[34] | 8 |
|  | Conduct educational outreach visits | - | - |
|  | Create a learning collaborative (II) | - Information meetings with external facilitators, an information leaflet was distributed[32] | 1 |
|  | Distribute educational materials (I) | - Distributed leaflet to the patients[30] - Distribute the ‘CSNAT training’ toolkit[26] - IPOS clinical guideline was distributed[32] | 3 |
|  | Develop educational materials (I) | - Facilitation _ Development of the ‘CSNAT training’ toolkit[26] | 1 |
|  | Make training dynamic (I) | - Implementation _ Stepwise introduce[15] - Education about the measure and use of results[33] - Training sessions regarding IPOS (flexible)[32] | 3 |
|  | Provide ongoing consultation (I) | - Facilitators _ Continuous consultation and peer support[26] - Conduct ongoing consultation[32] | 2 |
|  | Use train-the-trainer strategies (I) | - Trained the unit director to be the trainer in the service[31] - Facilitation _ Trained the IFs[26] | 2 |
|  | Work with educational institutions | - | - |
|  | Shadow other experts | - | - |
|  | **Sum strategies** | **24** | |
| **Support clinicians** | Create new clinical teams (III) | - Support from a restructured consultant roster[27] - Integration the supportive services for patient care[29] - Establishment of a Screening Committee[29] | 2 |
|  | Develop resource sharing agreements (III) | - Education offered by the PCOC team[27] - Facilitation _ External facilitators-Members of the CSNAT team[26] - External facilitators[32] - External support[34] | 4 |
|  | Facilitate relay of clinical data to providers (I) | - Using results in real time[31] - Daily pain assessment and specialist referral in time[30] - Inner setting _ Utilisation in daily interactions[15] - Education about the measure and use of results[33] - Use the assessment result at daily handover[27] - Use the assessment results[32] - Assessment handling[29] - Enable sensitive use[34] | 8 |
|  | Revise professional roles (III) | - Context _ Organisation culture[26] - Defining who applies the measure to which patients[31] - Empowering the PC team to use POS and Modified POS-S results[31] | 2 |
|  | Remind clinicians (II) | - Reminders[25] - Internal facilitation process _ Continuous communication[26] - Reminder system[29] - Reminder system[28] | 4 |
|  | **Sum strategies** | **22** | |
| **Develop stakeholder interrelationships** | Build a coalition (I) | - Inner setting _ Team working[15] - Facilitators _ Continuous consultation and peer support[26] - Clarity the IFs roles _ Support from co-facilitators[26] - Collaborative approach: bring others on board[26] | 2 |
|  | Conduct local consensus discussions (I) | - Evidence _ Informed advocacy: ability to distinguish intervention from existing practice[26] - Understanding and discussing difficulties via meeting _ Importance of using a measure routinely[31] | 2 |
|  | Capture and share local knowledge | - | - |
|  | Develop an implementation glossary | - | - |
|  | Develop academic partnerships | - | - |
|  | Inform local opinion leaders (I) | - Clarity the IFs roles _ Authority to make change [26] | 1 |
|  | Involve executive boards (II) | - Improving the quality of the implementation through data entry and data review[27] - Context _ Leadership support[26] - Leadership support[32] - Stakeholder and leadership engagement[28] | 4 |
|  | Identify and prepare champions (I) | - Identity a champion[25] - Implementation _ Champions & facilitators[15] - Identify the Internal facilitator_ Position of IF: embeddedness within implementation team[26] - Identify internal facilitators[32] | 4 |
|  | Identify early adopters | - | - |
|  | Model and simulate change (II) | - Model for pilot study and modified during the post-pilot period[31] - Modelling the intervention[30] | 2 |
|  | Organize clinicians implementation team meetings (I) | - Understanding and discussing difficulties via meeting[31] - Team meetings[27] | 2 |
|  | Obtain formal commitment | - | - |
|  | Promote network weaving | - | - |
|  | Recruit, designate, and train for leadership (IV) | - Provide project support[25] - Inner setting _ Support leadership[15] | 2 |
|  | Use advisory boards and workgroups | - | - |
|  | Use an implementation advisor | - | - |
|  | Visit other sites | - | - |
|  | **Sum strategies** | **21** | |
| **Use evaluative and iterative strategies** | Assess for readiness and identify barriers and facilitators (I) | - Identify barriers and opportunities, and tailor the implementation strategy to the context[32] | 1 |
|  | Audit and provide feedback (I) | - Ongoing feedback[25] - External audit[30] - Implementation _ Feedback[15] - Analyses of outcome measures over time[33] - Audit and feedback[27] - Internal facilitation process _ Proactive problem solving[26] - Provide feedback[32] - Understanding and discussing difficulties via meetings[31] - Refine the workflow through iterative feedback[29] - Continuous feedback and adjustment[34] | 10 |
|  | Conduct local needs assessment (I) | - Selection of outcomes of interest[33] | 1 |
|  | Conduct cyclical small tests of change (I) | - Small scale changes[27] | 1 |
|  | Develop and implement tools for quality monitoring (I) | - Assessing the quality of the intervention by setting indicators on[30] - Improving the quality of the implementation through data management[27] | 2 |
|  | Develop and organize quality monitoring systems | - | - |
|  | Develop a formal implementation blueprint (I) | - A service-level implementation strategy[25] | 1 |
|  | Purposively re-examine the implementation | - | - |
|  | Obtain and use patients/consumers and family feedback | - | - |
|  | Stage implementation scale up (I) | - Pilot test before the formal implementation[31] - Pilot implementation of the intervention[30] - Pilot test[15] - Pilot test before the formal implementation[29] | 4 |
|  | **Sum strategies** | **20** | |
| **Adapt and tailor to context** | Promote adaptability (I) | - Choosing PCOMs suitable for the service[31] - Adjust the intervention more flexible [25] - Appropriateness and validity of PCOMs in palliative care[15] - Selection of outcome measure(s)[33] - Selection and modification of PCOMS[29] - Intervention developed based on the assessment[29] - ePRO selection[28] | 6 |
|  | Tailor strategies (I) | - Analysing the local context (professionals and patients)[30] - Identify barriers and opportunities, and tailor the implementation strategy to the context[32] - Clinical workflow development[28] | 3 |
|  | Use data experts | - | - |
|  | Use data warehousing techniques | - | - |
|  | **Sum strategies** | **10** | |
| **Provide interactive assistance** | Centralize technical assistance (III) | - Inner setting _ Healthcare environment[15] | 1 |
|  | Facilitation (I) | - Internal facilitation process[26] - Internal facilitators roles[32] | 2 |
|  | Provide local technical assistance (IV) | - Context _ Establish a carer record with IT staff[26] - Inner setting _ IT infrastructure[15] - Records integrated into the electronic databases[33] - Electronic Health Record (EHR) Integration[29] - IT continuous support[29] - EHR integration and data security[28] - IT support[34] | 6 |
|  | Provide clinical supervision | - | - |
|  | **Sum strategies** | **10** | |
| **Change infrastructure** | Change accreditation or membership requirements | - | - |
|  | Change record systems (III) | - Context _ Establish a carer record with IT staff[26] - An electronic device was used[30] - Records integrated into the electronic databases[33] | 3 |
|  | Change service sites | - | - |
|  | Change liability laws (III) | - Evidence _ Legitimising the intervention to change practice[26] | 1 |
|  | Change physical structure and equipment | - | - |
|  | Create or change credentialing and/or licensure standards | - | - |
|  | Mandate change | - | - |
|  | Start a dissemination organization | - | - |
|  | **Sum strategies** | **4** | |
| **Utilize financial strategies** | Access new funding | - | - |
|  | Alter incentive/allowance structures (III) | - Outer setting _ Policy driver[15] | 1 |
|  | Alter patient/consumer fees | - | - |
|  | Develop disincentives | - | - |
|  | Fund and contract for clinical innovation (IV) | - Outer setting _ Funding[15] | 1 |
|  | Make billing easier | - | - |
|  | Place innovation on fee for service lists/formularies | - |  |
|  | Use other payment schemes | - | - |
|  | Use capitated payments | - | - |
|  | **Sum strategies** | **2** | |
| **Engage consumers** | Increase demand |  |  |
|  | Involve patients/consumers and family members (I) | - Education for both patients and nonprofessional caregivers[30] - Patient onboarding[28] | 2 |
|  | Intervene with patients/consumers to enhance uptake and adherence | - | - |
|  | Prepare patients/consumers to be active participants | - | - |
|  | Use mass media | - | - |
|  | **Sum strategies** | **2** | |
|  | **38/73** | **115** | |

**Note:** I, II, III, and IV are the Go-zone quadrants of the ERIC implementation strategies, which divided based on the importance and feasibility ratings of the implementation strategies[18]. Quadrant I = implementation strategy with the highest levels of importance and feasibility, quadrant II = strategies were relatively high in feasibility, quadrant III = strategies were low in importance and feasibility, and Quadrant IV = strategies were relatively high in importance.

**Supplementary table 4 Meta themes synthesized from the identified implementation strategies**

| **Meta themes** | | **Identified ERIC implementation strategies in the included studies** | **Categories of strategies in ERIC** |
| --- | --- | --- | --- |
| **What to do** | -Build capacity  -Develop and share resources through training and education  -Establish stakeholders relationships  -Support palliative care professionals  -Change infrastructure both at the organizational and cultural level | -Conduct ongoing training[15, 27, 30, 34]  -Conduct educational meetings[25, 27-30, 32-34]  - Create a learning collaborative[32]  - Distribute educational materials[26, 30, 32]  - Develop educational materials[26]  - Make training dynamic[15, 32, 33]  - Provide ongoing consultation[26, 32]  - Use train-the-trainer strategies[26, 31] | Train and educate stakeholders |
|  |  | - Build a coalition[15, 26]  - Conduct local consensus discussions[26, 31]  - Inform local opinion leaders[26]  - Involve executive boards[26-28, 32]  - Identify and prepare champions[15, 25, 26, 32]  - Model and simulate change[30, 31]  - Organize clinicians implementation team meetings[27, 31]  - Recruit, designate, and train for leadership[15, 25] | Develop stakeholder interrelationships |
|  |  | - Create new clinical teams[27, 29]  - Develop resource sharing agreements[26, 27, 32]  - Facilitate relay of clinical data to providers[15, 27, 30-33]  - Revise professional roles[26, 31]  - Remind clinicians[25, 26, 28, 29] | Support clinicians |
|  |  | - Change record systems[26, 30, 33]  - Change liability laws[26] | Change infrastructure |
| **How to do it** | -Be agile, responsive, and interactive in the supported provided | - Assess for readiness and identify barriers and facilitators[32]  - Audit and provide feedback[15, 25-27, 29-34]  - Conduct local needs assessment[33]  - Conduct cyclical small tests of change[27]  - Develop and implement tools for quality monitoring[27, 30]  - Develop a formal implementation blueprint[25]  - Stage implementation scale up[15, 29-31] | Use evaluative and iterative strategies |
|  |  | - Promote adaptability[15, 25, 28, 29, 31, 33]  - Tailor strategies[28, 30, 32] | Adapt and tailor to context |
|  |  | - Centralize technical assistance[15]  - Facilitation[26, 32]  - Provide local technical assistance[15, 26, 28, 29, 33, 34] | Provide interactive assistance |
|  |  | - Alter incentive/allowance structures[15]  - Fund and contract for clinical innovation[15] | Utilize financial strategies |
| **Who to do it with** | -Involved all the relevant stakeholders, including palliative care professionals, patients, family/carers, those who providing education and/or implementation guidance, hospital administrative leaders/staff, and those involved in funding services | See the above information | Train and educate stakeholders |
|  |  |  | Develop stakeholder interrelationships |
|  |  |  | Support clinicians |
|  |  |  | Change infrastructure |
|  |  |  | Use evaluative and iterative strategies |
|  |  |  | Adapt and tailor to context |
|  |  |  | Provide interactive assistance |
|  |  |  | Utilize financial strategies |
|  |  | - Involve patients/consumers and family members[28, 30] | Engage consumers |
